# Post-Transcriptional Dysregulation of microRNA and Alternative Polyadenylation in Colorectal Cancer

**DOI:** 10.3389/fgene.2020.00064

**Published:** 2020-02-21

**Authors:** Zhanrui Mao, Hui Zhao, Yulan Qin, Jianming Wei, Jielin Sun, Weiwei Zhang, Yani Kang

**Affiliations:** ^1^ School of Biomedical Engineering, Bio-ID Center, Shanghai Jiao Tong University, Shanghai, China; ^2^ Department of General Surgery, Tianjin Medical University General Hospital, Tianjin, China; ^3^ Shanghai Center for Systems Biomedicine, Shanghai Jiao Tong University, Shanghai, China; ^4^ State Key Laboratory of Oncogenes and Related Genes, Renji-Med X Clinical Stem Cell Research Center, Ren Ji Hospital, School of Medicine, Shanghai Cancer Institute, Shanghai Jiao Tong University, Shanghai, China

**Keywords:** microRNAs, colorectal cancer, alternative polyadenylation, posttranscriptional regulation, 3'UTR

## Abstract

**Background:**

Colorectal cancer (CRC) is one of the leading causes of cancer death worldwide. microRNAs (miRNAs) repress gene expression by binding to complementary sequences in the 3' untranslated region (3'UTR) of target mRNAs. Alternative polyadenylation (APA) are relevant to the variability of the 3'UTR of mRNA. However, the posttranscriptional dysregulation of miRNAs and APA in CRC are poorly understood.

**Method:**

In this study, we conducted small RNA sequencing to identify differentially expressed miRNAs (DERs) and their target genes. Function analysis on DER-target genes can explain the regulation roles of miRNAs in CRC. The mutual regulation of miRNAs and APA was analyzed by combining miRNA data to 3'UTR alteration using 3' termini of polyadenylated RNAs sequencing (3T-seq) technique, and this was validated using TCGA gene expression data.

**Results:**

Our results showed 64 significant differentially expressed miRNAs (DERs) in CRC patients. Their target genes were related to cell adhesion and transcription regulation and were prevailingly involved in the CRC-related pathway. Integrative analysis of the miRNA and APA profile revealed 16 DERs were correlated with 12 polyadenylation factors, and six of them were significantly differently expressed in CRC. We also found four DERs that lost binding sites due to APA and showed a positive correlation between the miRNA and gene expression.

**Conclusion:**

Our study found that miRNAs regulated APA by modulating key polyadenylation factors, and several miRNAs lost their suppression on mRNA due to APA. Associating this with gene expression may provide some important clues for a deeper study of posttranscriptional cellular regulation and biomarker research in CRC. Our data provided the first evidence that the interaction between miRNAs and APA associated with gene expression could serve as biomarkers for CRC, suggesting that hsa-miR-133a-3p and *MLEC*, hsa-miR-145-5p and *SET*, hsa-miR-1-3p and *PPIA*, and hsa-miR-378d and *YY1* might be novel and potential biomarkers in improving the diagnosis of CRC.

## Introduction

Colorectal cancer (CRC) is the third most deadly cancer globally and remains among the top five of all cancers in China in terms of mortality (Cunningham et al., 2010; Rawla et al., 2019). The classic description of CRC tumorigenesis is the accumulation of genetic alterations in defined oncogenes and tumor suppressor genes which results from chromosomal instability (CIN) and microsatellite instability (MSI) (Lynch and de la Chapelle, 2003). CIN is proven to be mechanism of genomic instability in majority of colon cancers, which leads to a different pattern of gene alterations contributing to tumor progress. The most known genetic alterations include the activation of oncogenes such as *KRAS* and the inactivation of tumor suppressor genes such as *TP53*, *SMAD2*, *TGFBR2,* and *APC* (Lynch and de la Chapelle, 2003).

miRNAs are a group of ~22-nucleotide small noncoding RNAs that mediate posttranscriptional gene silencing. miRNAs repress gene expression by binding to complementary sequences in the 3′ untranslated region (3′ UTR) of mRNAs to target them for degradation and thereby prevent their translation. Increasing numbers of evidence indicates that miRNAs regulate oncogenes and tumor suppressor gene expressions. They target the signaling pathways by aﬀecting important factors of CRC development and malignancy, such as EGFR/KRAS, EGFR/mTOR, TGFβ, p53, and EMT transcription factors (Wang et al., 2015). Several studies have indicated deregulations of some known tissue-specific miRNAs, e.g., let-7, miR-9, miR-17, miR-19, miR-21, miR-24, and miR-155 in CRC patients, which could be used as potential diagnostic and therapeutic biomarkers in CRC patients (Moridikia et al., 2018).

Alternative polyadenylation (APA) is an important aspect of posttranscriptional regulation and is considered as a fundamental mediator of gene expression involved in many types of cancers, including CRC (Elkon et al., 2013). During the usage of the alteration of polyadenylation sites, a shorter 3'UTR is generated by choosing the polyadenylation site (PAS) that was most proximal to the translated region. This eliminates miRNA-binding sites and makes the mRNA lose the suppression effect of miRNA (Lin and Gregory, 2015). Meanwhile, miRNA dysregulation affects APA by targeting key polyadenylation factors (Zhu et al., 2016). However, few studies have reported the interaction between miRNAs and APA in CRC.

In this study, we combined small RNA sequencing and 3' termini of polyadenylated RNAs sequencing (3T-seq), our previously developed and published method (Lai et al., 2015), in tumor tissue and paired normal tissue of CRC patients for the first time. The alteration of polyadenylation sites on the 3'UTR of differentially expressed miRNAs (DERs) target genes that related to cancer and the effect of the miRNA regulation of APA factors in CRC were analyzed to understand gene expression dysregulation in CRC at the posttranscriptional level.

## Materials and Methods

### Collection of Human Tissue Samples

Fresh tissue samples from CRC patients and matched normal tissues were collected at the Renji Hospital of Shanghai Jiao Tong University. This study was approved by the Institutional Review Boards of Shanghai Jiao Tong University School of Medicine, Renji Hospital Ethics Committee (RA-2019-316) and carried out in accordance with the Code of Ethics of the World Medical Association (Declaration of Helsinki). All patients signed an informed consent form. All samples were examined by one experienced pathologist and the clinical information of all individuals is listed in **Table S1**.

After collection, samples were quickly put in liquid nitrogen and preserved in a low temperature environment. TRIzol (Invitrogen, Waltham, Massachusetts, USA) was used to isolate total RNA from tissue samples following the manufacturer's protocol. The total RNA quantity was determined with Nanodrop 2000 (Thermo Scientific, Waltham, Massachusetts, USA) and quality was assessed by running 1% agarose gel electrophoresis, using 4S Red Plus Nucleic Acid Stain (Sango, Shanghai, China).

### miRNA-Seq Analysis

miRNA-seq libraries were prepared using the TruSeq^®^ Small RNA Library Preparation kit from Illumina^®^ and sequenced at the Bio-ID Center, China, using a 50-bp single-end reads Illumina NextSeq 500 Sequencer (Illumina Inc., San Diego, CA, USA). Raw sequence data was submitted to the Gene Expression Omnibus (GEO) (accession numbers: GSE130084). After sequencing, adapters and low-quality sequences were removed from obtained raw reads and clean data was counted using the Fastqc program. 15–30 nt fragments were reserved for subsequent analysis and mapped to the reference genome using the Bowtie2 program.

### miRNAs Identification and Expression Analysis

miRNA-seq clean data was compared to the known miRNA database in miRBase using the Bowtie2 program. miRDeep2 was used to predict novel miRNA. miRNA expression levels were normalized to RPM (reads per million). Differentially expressed known and novel miRNA were calculated using edgeR. The miRNAs with absolute value of Log_2_ fold changes ≥1, RPM ≥ 0.1, and a *p* value ≤ 0.05 were considered DERs.

### Target Genes Prediction and Functional Analysis

Target genes of DERs were predicted using miRTarBase (release 7.0) (Chou et al., 2018). The gene expression analysis for top 50 DERs target genes with largest number of gene-miRNA interactions, which is greater than 15 was performed on RNA-seq data of 275 CRC patients and 41 matched normal samples from TCGA project using GEPIA server (Tang et al., 2017). We used log2(TPM + 1) for log-scale. Target genes were then analyzed using the Gene Ontology (GO) database and the Kyoto Encyclopedia of Genes and Genomes (KEGG) pathway database for the functional annotation of the predicted DER-target genes in clusterProfiler (v3.10.1) with a *p* value less than 0.05 as a parameter.

### Construction of the DER-Target Genes Interaction Network

A miRNA-target genes interaction network was constructed based on the DERs and their target genes. The relationship between DERs and genes were identified using the STRING database (v11.0) (https://string-db.org/) (Szklarczyk et al., 2015). Finally, the interaction network of DERs and target genes was built by using the Cytoscape software (v2.8.3) by the threshold of 0.400 (Shannon et al., 2003).

### Integrative Analysis of CRC-Related DERs and APA-Mediated 3'UTR Alteration

The 3'UTR length change data between CRC tissues and paired normal tissues corresponding to DER-target genes was analyzed using previously published 3T-seq data (accession number: E-MTAB-6403) (Yang et al., 2018). Target and DER sequences were obtained in miRTarBase (release7.0) (Chou et al., 2018). The mutual regulation between CRC-related DERs and APA factors were validated by using RNA sequencing data between 275 colon adenocarcinoma tissue data points and 349 matched TCGA normal and GTEx tissue data points by GEPIA (http://gepia.cancer-pku.cn/) with a *p* value = 0.01 and |Log_2_ fold changes| = 1 as cut-off values (Tang et al., 2017).

## Results

### miRNA Profiles in CRC

Sequencing the miRNA libraries for *CRC* tissues and paired normal tissues resulted in a total of 105,820,744 and 143,340,789 raw reads, respectively. The removal of adaptor sequences, junk reads, reads other than 15 to 30 bp, rRNA, snRNA, snoRNA, and tRNA produced 96,341,699 and 119,318,139 clean reads, respected, and the valid reads that could be mapped to the reference genome were shown in **Table S2**. The clean reads accounted for no lower than 94% of the raw reads, which suggested that a useful group of miRNAs was obtained with a reasonable sequencing depth.

### Identification of Known and Novel miRNAs

After alignment, the lengths of valid reads ranged from 15 to 30 nt and the majority of reads converged on 20 to 24 nt, with 22 nt as the peak. This was consistent with previous reports (**Figure S1**). In total, we identified 1,286 miRNAs that perfectly matched known miRNAs using the criterion that average of reads should be greater than one in the same group. Five of them, including hsa-miR-143-3p, hsa-miR-26a-5p, hsa-miR-10a-5p, hsa-miR-10b-5p, and hsa-miR-192-5p, had above 50,000 RPM on average across all samples (**Table S3**). Then we used miRDeep2 algorithm to identify novel miRNAs. We also found 201 candidate miRNAs with lengths of 16–24 nt and average of reads greater than one in the same experimental condition (**Table S4**).

### CRC-Related DERs and Their Target Genes

**Figure 1:**
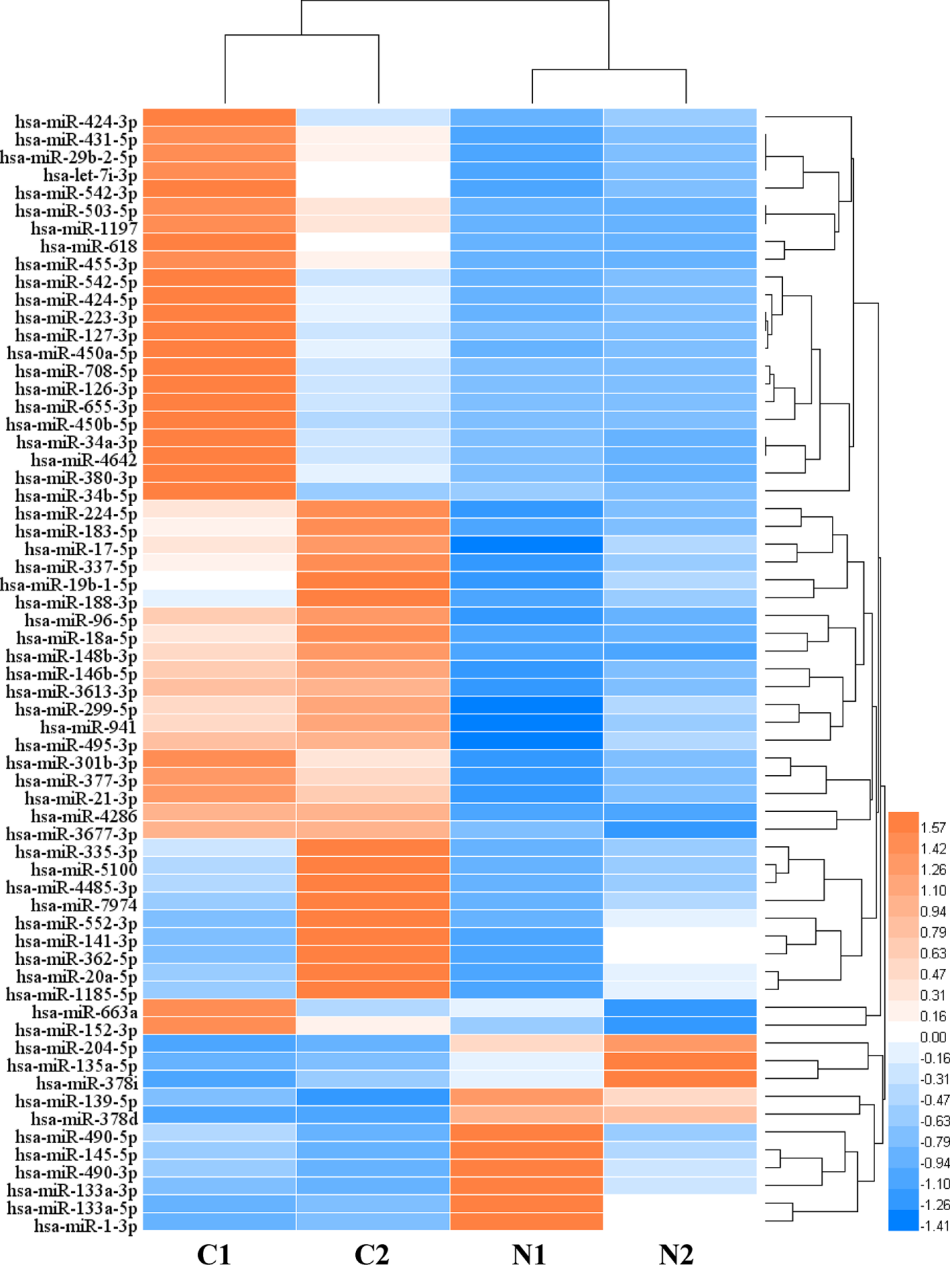
The heat map of differentially expressed microRNAs (miRNAs) (C: CRC tissue; N: paired normal tissue).

In order to analyze the significant miRNA in CRC patients, 64 differentially expressed miRNAs (DERs) were identified in the CRC patients under two conditions: *p* value < 0.05 and an absolute value of log_2_ fold change > 1. Then, to characterize the regulatory roles of CRC miRNAs, we identified 64 significant DERs (**Table S5**). 52 miRNAs were significantly upregulated and 12 were downregulated in CRC tissues, and the clustering analysis result indicated the miRNA differential expression patterns between CRC tissues and paired normal tissues (**Figure 1**). Among the 6,330 genes predicted as DERs targets, 5,984 genes were targeted by upregulated 52 miRNAs and 3,371 genes were targeted by downregulated 12 miRNAs (**Figure S2**).The results of expression analysis for top 50 genes with largest number of gene-miRNA interactions was shown in **Figure S3**. The top 5 most significantly overexpressed DERs target genes were *HMGA2*, *MYB*, *E2F1*, *MET,* and *CCNE2*. And the top five most significantly down-regulated DERs target genes were *ZEB2*, *BCL2*, *RECK*, *ZEB1,* and *ESR1*.

### Functional Analysis of DER-Target Genes

**Figure 2:**
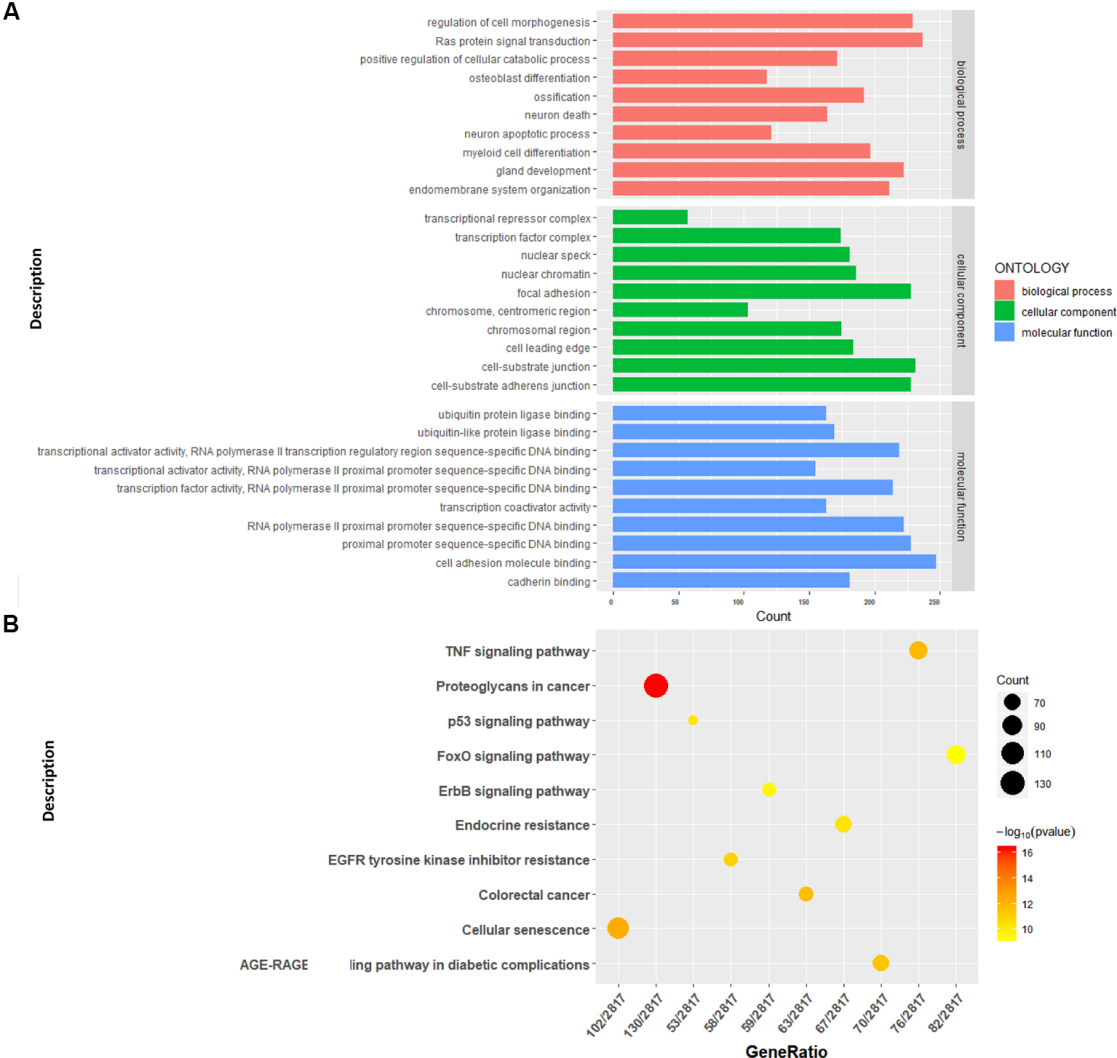
Functional analysis of the target genes of colorectal cancer (CRC)-related differentially expressed miRNAs (DERs). **(A)** Gene Ontology functional annotation of identified target genes. **(B)** Kyoto Encyclopedia of Genes and Genomes (KEGG) pathway annotation of identified target genes.

**Figure 3:**
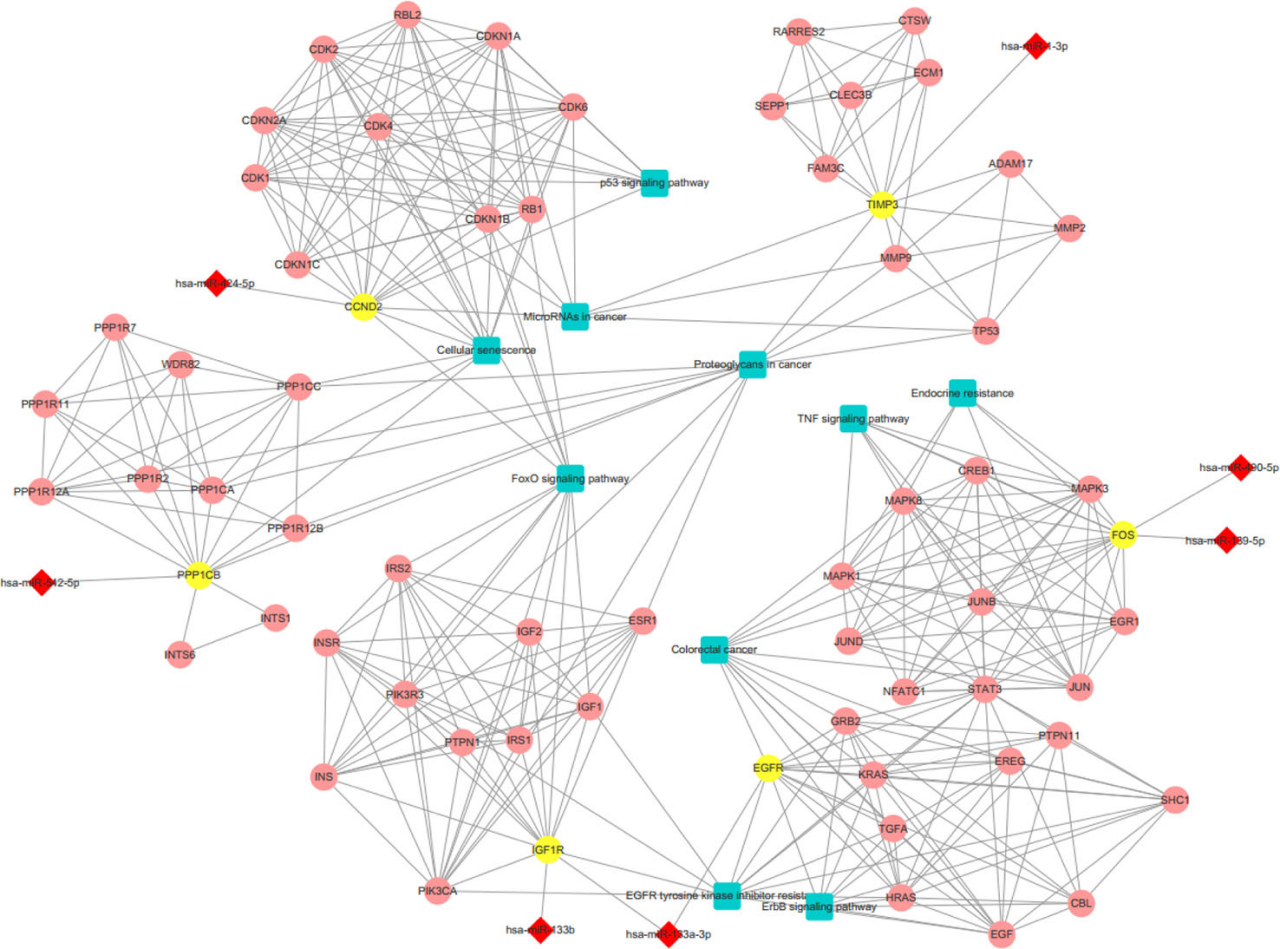
The interaction network of differentially expressed miRNAs (DERs) and genes. The red diamond represents DERs in colorectal cancer (CRC) patients. The yellow circle represents the DER-target gene. The pink circle represents the gene that interacts with the target gene. The blue rectangle represents the annotated pathway of the target gene.

We conducted a GO analysis on 6,330 DER-target genes to further analyze their functions in CRC, which resulted in the annotation of 138 GO terms (**Table S6**). The GO terms were significantly enriched in cell adhesion (GO: 0045296 cadherin binding; GO: 0050839 cell adhesion molecule binding), transcription regulation (GO: 0001228 transcriptional activator activity and RNA polymerase II transcription regulatory region sequence-specific DNA binding; GO: 0000978 RNA polymerase II proximal promoter sequence-specific DNA binding; GO: 0000987 proximal promoter sequence-specific DNA binding), and protein modifications (GO: 0044389 ubiquitin-like protein ligase binding; GO: 0019903 protein phosphatase binding; GO: 0019787 ubiquitin-like protein transferase activity) (**Figure 2A**). The KEGG pathway analysis showed that DER-target genes were prevailingly involved in the TNF signaling pathway, proteoglycans in cancer, the p53 signaling pathway, FoxO signaling pathway, and ErbB signaling pathway (**Figure 2B**). To further illustrate the regulatory functions of the CRC-related DERs and their target genes, a miRNA-target gene interaction network was constructed by Cytoscape (**Figure 3**). The network revealed that CRC-related DERs such as hsa-miR-1-3p, hsa-miR-490-3p, hsa-miR-542-5p, hsa-miR-424-5p, and hsa-miR-133a-3p are associated with the regulation of several cancer-related pathways by targeting *TIMP3*, *FOS*, *PPP1R12B*, *CCND2,* and *EGFR*.

### miRNAs Regulate APA by Targeting Polyadenylation Factors in CRC Patients

**Figure 4:**
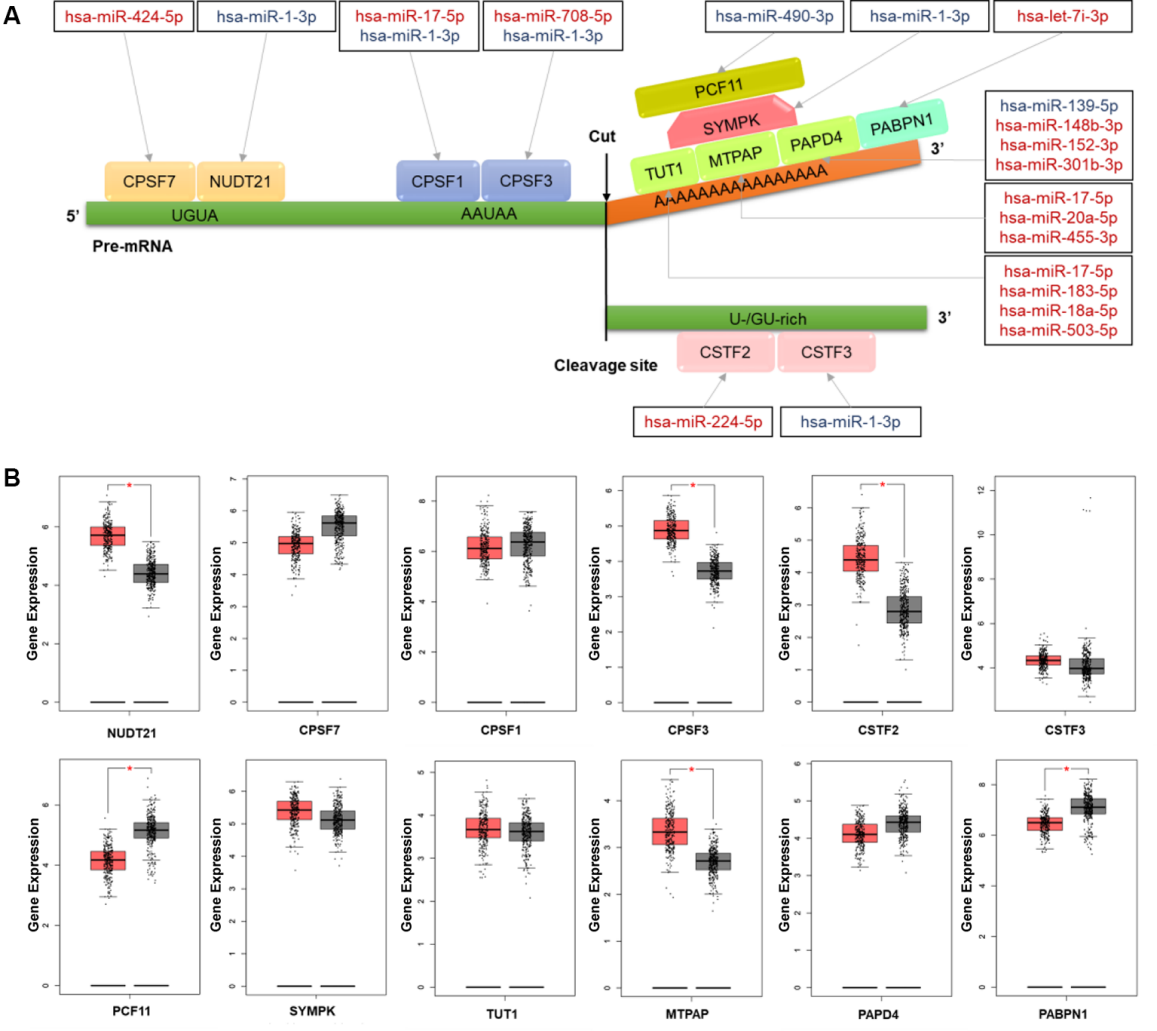
The colorectal cancer (CRC)-related differentially expressed miRNAs (DERs) and their targets involving the mechanism of alternative polyadenylation (APA) **(A)** Schematic diagram of microRNAs (miRNAs) and their target factors involving the polyadenylation mechanism in CRC. Upregulated miRNAs are marked in red and downregulated miRNAs are marked in blue. **(B)** The box plot of polyadenylation factor expression in CRC tissues (red) and normal tissues (grey). The expression data were log_2_(TPM+1) transformed for differential analysis and the log_2_FC was defined as median (CRC tissues) – median (normal tissue). *p value <0.05.

To understand the regulation roles of miRNAs in APA mechanisms, we analyzed the relationships between CRC-related DERs and key cleavage and polyadenylation factors. In total, we found 16 DERs were correlated with 12 polyadenylation factors involved in multiprotein complexes such as cleavage and polyadenylation specific factor (CPSF), cleavage stimulation factor (CSTF), cleavage and polyadenylation factor, symplekin protein, poly(A) binding protein (PABP), and poly(A) polymerase(PAP) (**Figure 4A**). Furthermore, hsa-miR-1-3p was found to regulate five CPSFs, including *NUDT21*, *CPSF1*, *CPSF3*, *SYMPK,* and *CSTF3*. Thus, its role in APA regulation may be noteworthy. Detailed information on all these APA factors are shown in **Table S7**. Through the validation of using the gene expression of CRC patients from TCGA RNA-seq data, the expression of 6 out of 12 key cleavage and polyadenylation factors were significantly different in tumor tissues. *NUDT21*, *CPSF3*, *CSTF2,* and *MTPAP* were overexpressed while *PCF11* and *PABPN1* were suppressed in colon cancer tissues (**Figure 4B**). Subsequently, the expression change of all 6 aforementioned genes results were confirmed in C1 and C2 samples comparing to their corresponding adjacent samples (**Figure S4**). And the *NUDT21*, *CPSF3*, *CSTF2*, and *MTPAP* were overexpressed and the expression of *PCF11* and *PABPN1* were decreased in C1 and C2 samples comparing to their corresponding adjacent samples.

### APA-Meditated 3'UTR Alteration Leads to miRNA Binding Site Elimination in CRC Patients

**Figure 5:**
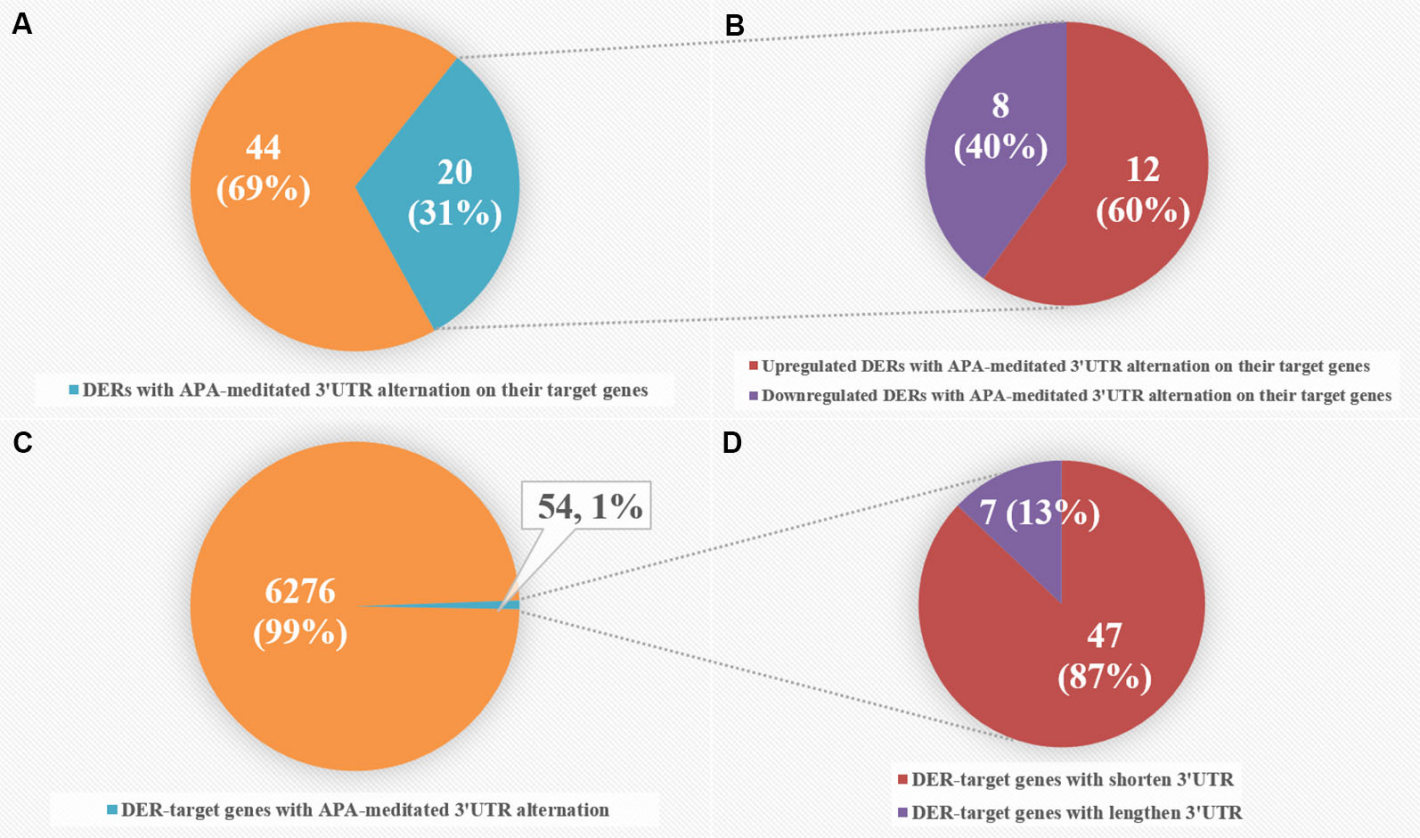
Comparative analyses of differentially expressed miRNAs (DERs) and DER-target genes with alternative polyadenylation (APA)-meditated 3'UTR alteration in colorectal cancer (CRC) patients. **(A)** The number of DERs with APA-meditated 3'UTR alteration on their target genes. **(B)** The number of upregulated and downregulated DERs with APA-meditated 3'UTR alteration on their target genes. **(C)** The number of DER-target genes with APA-meditated 3'UTR alteration. **(D)** The number of DER-target genes with shortened and lengthened 3'UTRs.

To intensively explore the relationship between APA and the expression of CRC-related DERs and their target genes, we combined miRNA-seq data with our previously published 3T-seq data of CRC patients, which specifically enriched 3' termini of RNAs and adopted 3'UTR length index (CULI) to quantitatively characterize the 3'UTR alteration. Comparing the APA change with mRNA abundance, we found 54 target genes of 20 miRNAs with consistent 3'UTR alterations in all CRC patients (**Table S8**). As shown in **Figure 5**, APA-mediated target gene 3'UTR alterations appeared in 31% of CRC-related DERs, with 40% downregulated and 60% upregulated in CRC patients. However, DER-target genes with APA-mediated 3'UTR alteration merely accounted for 1% of all 6,330 genes that were predicted as target genes of CRC-related DERs, while 13% of these genes had lengthened 3'UTRs and 87% of them had shortened 3'UTRs.

**Figure 6:**
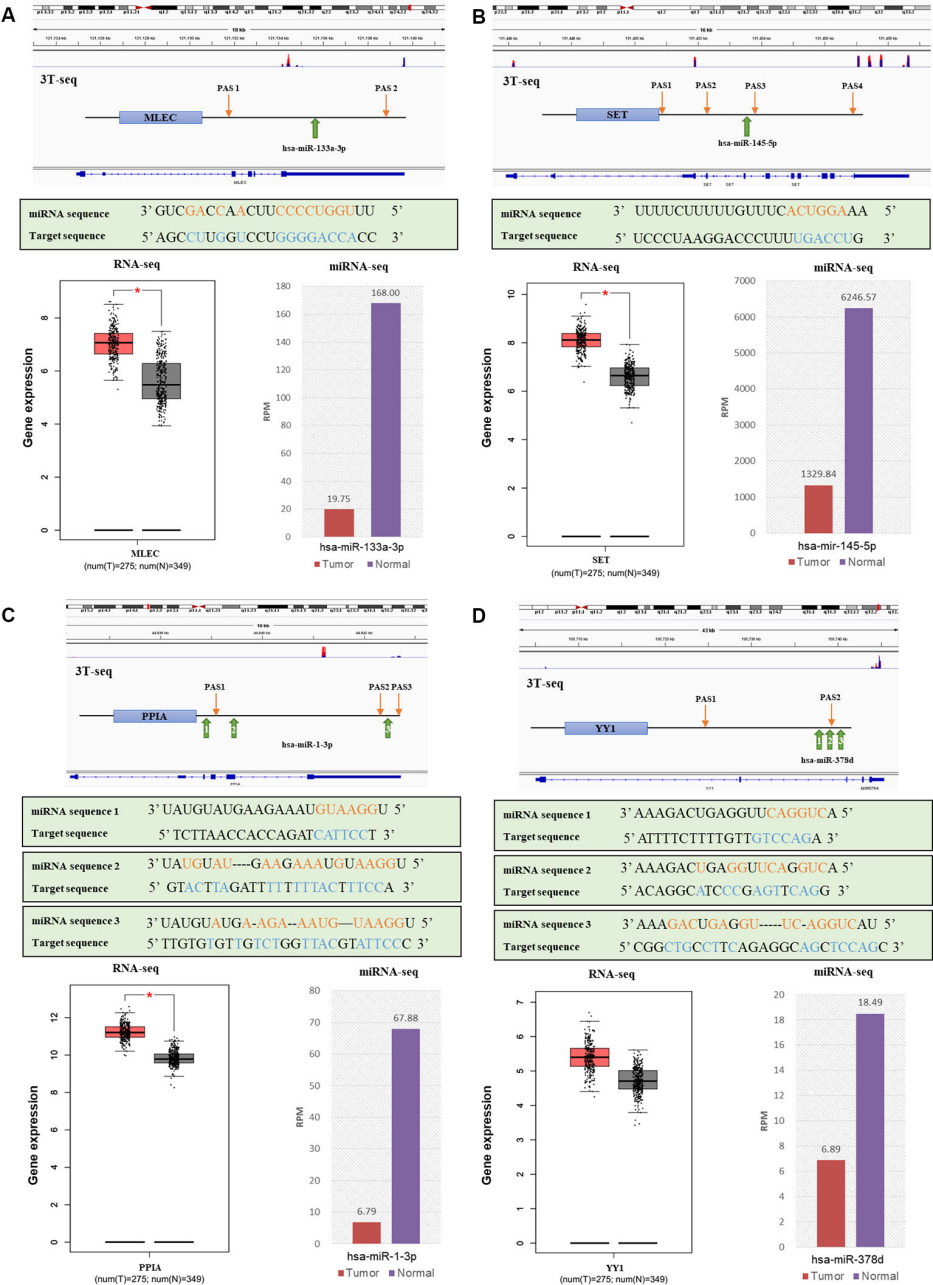
The relationships of alternative polyadenylation (APA) alteration and microRNA (miRNA) binding sites lost with miRNA downregulation and target gene upregulation. **(A)** *MLEC* and hsa-miR-133a-3p. **(B)** *SET* and hsa-miR-145-5p. **(C)** *PPIA* and hsa-miR-1-3p. **(D)** *YY1* and hsa-miR-378d. Each figure, from top to bottom are a genomic view of gene transcript isoforms with alternative poly(A) sites in colorectal cancer (CRC) tissues (overlay in red) and normal counterparts (overlay in blue); the schematic diagram of polyadenylation sites (PASs) relative location and miRNA binding sites of each gene; the miRNA mature sequence and the target sequence of each binding site, where complementary base are labeled orange and blue; the box plot of gene expression in CRC tissues (red) and normal tissues (grey); and the histogram of miRNA expression in CRC tissues (red) and normal counterparts (purple).

Then, to obtain the relationship between APA-mediated DER-target gene 3'UTR alterations and gene expression, we analyzed the relative location of alternative polyadenylation sites and DER binding sites. We filtered out four downregulated miRNAs that lost binding sites due to APA-meditated 3'UTR shortening of their target genes accompanied by mRNA upregulation in CRC patients (**Table S9**). Data from 3T-seq that was exhibited on Integrative Genomics Viewer (IGV) software showed gene transcript isoforms with alternative poly(A) sites in CRC tissues (overlay in red) and normal counterparts (overlay in blue) on the top. The schematic diagram representing the PASs relative location and miRNA binding sites of each gene were in the second place. The box plot and histogram showed gene and miRNA expression, respectively, in CRC tissues (T) and normal tissue (N). For example, hsa-miR-133a-3p was the most significantly downregulated miRNA in CRC patients and *MLEC*, as its target gene, preferentially used proximal PAS in CRC patients. Therefore, there was a lost target site of hsa-miR-133a-3p in *MLEC* 3'UTR, which was located right between the distal PAS and proximal PAS. Similarly, other miRNA and their target genes—hsa-miR-145-5p and *SET*, hsa-miR-1-3p and *PPIA*, and hsa-miR-378d and *YY1*—all shared the same pattern as hsa-miR-133a-3p and *MLEC*. 3'UTR was shortened, resulting in the loss of miRNA binding sites. Except for *YY1*, the analysis of TCGA RNA-seq data indicated the significant overexpression of *MLEC*, *SET,* and *PPIA* in 275 CRC patients (**Figure 6**). We have also applied the survival analysis by using ENCORI (The Encyclopedia of RNA Interactomes) platform based on TCGA project data (Li et al., 2013). The survival analysis results showed the high expression of hsa-miR-133a-3p, hsa-miR-1-3p, hsa-miR-378d, and YY1 correlated to low overall survival rate, but the correlations were not statistically significant (**Figure S5**).

## Discussion

MiRNAs are a class of ~22-nucleotide noncoding small RNAs that play important roles in mediating posttranscriptional gene silencing. MiRNAs repress gene expression by binding to complementary sequences in the 3'UTR of mRNAs, targeting them for degradation and thereby preventing their translation. The net effect of widespread miRNA depletion was to promote tumorigenesis. 3'UTR shortening could increase gene expression level by eliminating miRNA- binding sites (Wang et al., 2015). Identification and characterization of meaningful miRNAs, gene modules, and representative biomarkers related to the CRC could contribute to cancer diagnosis and the reveal of mechanisms behind cancer development.

In this study, the DER-target genes were significantly enriched in cell adhesion, transcription regulation, and protein modifications and were prevailingly involved in the p53 signaling pathway, which is an important cancer-related pathway. Previous studies have found that p53, encoded by the *TP53* gene, was one of the most important tumor suppressors that is frequently inactivated in gastrointestinal cancer. miRNAs have recently been recognized as mediators and regulators of p53 signaling (Wang et al., 2015).Our data showed that, as regulators of *TP53*, hsa-miR-18a-5p and hsa-miR-20a-5p were significantly up-regulated in CRC patients. The miRNA gene regulation network also revealed several CRC-related DERs, such as hsa-miR-1-3p, hsa-miR-133b, hsa-miR-133a-3p, hsa-miR-139-5p, hsa-miR-424-5p, hsa-miR-490-5p, and hsa-miR-542-5p, are associated with the regulation of CRC by targeting *TIMP3*, *FOS*, *IGF1R*, *PPP1R12B*, *CCND2,* and *EGFR*.

 APA is a common mechanism involving the structure of the 3'UTR, which is gaining increasing interest in cancer research. Approximately 70% of known human genes have multiple poly(A) sites in their 3'UTRs, and the variability of 3' untranslated regions of mRNA affect miRNA–mRNA interactions (Derti et al., 2012). Still, the knowledge of the mutual regulation of miRNAs and APA in CRC is still insufficient. The mechanism of polyadenylation includes two steps: mRNA cleavage by polyadenylation machinery proteins and the addition of a poly (A) tail *via* polyadenylation polymerases (Erson-Bensan and Can, 2016). The effects of miRNA on polyadenylation machinery factors are possible inducers of the selection of alternate poly (A) signals on the pre-mRNA that leads to the generation of isoforms with various lengths. This should be taken into consideration to better understand the extent and mechanisms of deregulated APA in cancer.

This is the first time the relationship between miRNA regulation and mRNA alternative polyadenylation has been explored, and this was done by combining miRNA profile and global polyadenylation site data from 3T-seq of CRC patients reported in our previous study (Yang et al., 2018). We found 16 DERs targeting 12 polyadenylation factors in total. It is interesting that, for some polyadenylation factors, the expression changes of miRNAs seem inconsistent with the expression change of their targets, including *CSTF2*, *PCF11,* and *MTPAP*. This may suggest that other regulatory mechanisms are also involved in the regulation of polyadenylation, along with miRNA. It is also noteworthy that other factors, such as *NUDT21* and *PABPN1*, have already been determined as APA regulators. *Nudt21* was shown to direct differential polyadenylation and, when suppressed, delayed progenitor cell differentiation (Brumbaugh et al., 2018). The modulation of *NUDT21* expression induced global APA alteration, and the knockdown of *NUDT21* increased the usage of the proximal polyadenylation site in serval tumor suppressor genes and promoted cell proliferation, metastasis, and tumorigenesis in liver cancer (Brumbaugh et al., 2018; Tan et al., 2018). *PABPN1* was viewed as a suppressor of APA, and the downregulation of *PABPN1* resulted in extensive 3'UTR shortening (Jenal et al., 2012). However, the role of *PABPN1* in cancer progression and development remains unknown. Some of the miRNA-target interactions we screened in CRC patients were identified previously *via* other experiments. For example, the interaction between hsa-miR-17-5p and *CPS1* was revealed in human embryonic kidney 293 cells by Helwak A, et al. using transcriptome-wide CLASH (crosslinking, ligation, and sequencing of hybrids) method (Helwak et al., 2013). And the interaction between hsa-miR-17-5p and *CPS1* and the interaction between hsa-miR-17-5p and *CPS3* were validated in HeLa cells by Selbach M, et al. through proteomic approach SILAC (stable isotope labelling with amino acids in cell culture) (Selbach et al., 2008). Indeed, functional analysis of miRNA-target interaction was also important in the in-depth research posttranscriptional dysregulation of miRNAs and APA in cancer and the relative reports were still limited in CRC. It had been found by Zhu Z J, et al. in osteosarcoma cells that hsa-miR-181a downregulated the expression of cleavage factor *NUDT21* and therefore inhibited the proliferation and promoted the apoptosis of the cells, which were confirmed by miRNA knockdown and cell culture experiments (Zhu et al., 2016).

The variability of 3' untranslated regions of mRNA caused by APA affects miRNA–mRNA interactions. Several studies have reported that global 3'UTR shortening was proven to be associated with cell transformation and cancers by eliminating miRNA-binding sites of cell growth control factors. This resulted in their escape of the suppression effect of miRNA-regulation and altered expression (Elkon et al., 2013; Lai et al., 2015; Masamha and Wagner, 2018). We found that APA-mediated 3'UTR alteration appeared in 54 DER-target genes and 20 corresponding miRNAs, accounting for 1% of all DER-target genes and 31% of all CRC-related DERs, respectively. Additionally, the majority of DER-target genes had shortened 3'UTRs in CRC patients, which is consistent with our former results that genes associated with cancer preferentially use proximal APA in CRC samples (Shannon et al., 2003). We also identified four miRNAs that lost binding sites of their target mRNA within 3'UTR. This was due to APA and showed a positive correlation between the miRNA binding sites lost and gene expression, including hsa-miR-133a-3p and *MLEC,* hsa-miR-145-5p and *SET*, hsa-miR-1-3p and *PPIA*, and hsa-miR-378d and *YY1.*The preferential cleavage at 3′UTR proximal poly(A) sites of these mRNA was observed, which was consistent with the previous report (Elkon et al., 2013).

*MLEC* is an endoplasmic reticulum-resident lectin participating in the identification and degradation of misfolding glycoproteins. Overexpression of *MLEC* was considered as one of the new markers in papillary thyroid carcinoma (Ban et al., 2012). Downregulation of hsa-miR-133a-3p has already been determined as a selective marker in colon cancer (Weber et al., 2018). Further study was needed to investigate the mechanism of hsa-miR-133a-3p suppression by targeting MLEC in CRC. *SET* is an important regulator of chromosome condensation and inhibitor of histone acetylation and active DNA demethylation (Almeida et al., 2017). A study of a gastric cancer cell line revealed that SET expression was positively correlated with cell proliferation and was associated with tumor progression and poor prognosis in human gastric cancer, and it may serve as a potential diagnostic marker (Yuan et al., 2017). Moreover, *SET* overexpression was proven to be a common alteration in early-stage CRC, promoting cell migration and EMT in CRC cells (Cristóbal et al., 2019). The miR-145-5p was downregulated in several tumors, including hepatocellular carcinomas, *via* p53 pathway apoptosis (Lupini et al., 2016). However, the posttranscriptional regulation role of miR-145-5p in CRC through *SET* has not yet been found.

*PPIA* has been reported to be upregulated in serval malignant tumors, like colorectal, pancreatic, lung, and hepatocellular carcinoma (Li et al., 2006; Gong et al., 2017; Nakano et al., 2017). The *PPIA* expression level also implied the clinical characteristics of resected lung adenocarcinomas and was correlated with a poor prognosis of lung adenocarcinoma (Nakano et al., 2017).However, the in-depth study of the *PPIA* overexpression mechanism in CRC was insufficient. Analysis of TCGA miRNA-sequencing data showed that downregulation of hsa-miR-1-3p may be a diagnostic marker of CR and that it was significantly related with the pathological stage (Zhou et al., 2018; Falzone et al., 2018). Therefore, further molecular biology experiments may be able to prove the interaction of the downregulation of hsa-miR-1-3p and the upregulation of *PPIA*, which is conducive to a better understanding of the CRC posttranscriptional regulation mechanism. *YY1* is a structural regulator of enhancer-promoter interactions and facilitates gene expression (Weintraub et al., 2017).The overexpression of *YY1* occurs in a range of cancer types, including lung, gastric, and colon cancer (Khachigian, 2018). In colon cancer cell lines, the ectopic expression of *YY1* stimulated cell growth and suppressed apoptosis, and it was targeted and inhibited by miR-7 (Zhang et al., 2013). However, the negative correlation of *YY1* and hsa-miR-378d has not been reported in CRC.

In conclusion, this study represented the first attempt to explore the posttranscriptional dysregulation of miRNA and APA of CRC. By combining miRNA profiles with global APA site states in CRC patients, we found miRNAs appeared to regulate APA by modulating APA regulators such as *NUDT21* and *PABPN1*, whereas APA regulated cancer-related gene expression by inducing 3'UTR alteration. This led to miRNA binding site elimination and its suppression effect termination in CRC patients, as was found in four miRNA-mRNA pairs. Additionally, our data provided the first evidence of the interaction between miRNAs and APA associated with gene expression involved in CRC, suggesting that hsa-miR-133a-3p and *MLEC*, hsa-miR-145-5p and *SET*, hsa-miR-1-3p and *PPIA*, and hsa-miR-378d and *YY1* might be novel and potential diagnostic biomarkers of CRC. It will improve our understanding of the underlying molecular mechanism of CRC in the posttranscriptional regulation level. However, our study still has limitations and further study is required. Future investigations are necessary to validate our results in a larger number of patients.

## Data Availability Statement

The miRNA-seq datasets generated for this study can be found in the Gene Expression Omnibus (GEO) (accession numbers: GSE130084). The 3T-seq datasets analyzed for this study can be found in the EMBL-EBI ArrayExpres (accession number: E-MTAB-6403).

## Ethics Statement

The studies involving human participants were reviewed and approved by Institutional Review Boards of Shanghai Jiao Tong University School of Medicine, Renji Hospital Ethics Committee (RA-2019-316) and was carried out in accordance with The Code of Ethics of the World Medical Association (Declaration of Helsinki). The patients/participants provided their written informed consent to participate in this study.

## Author Contributions

ZM designed the project, performed the sequencing and contributed to the draft of the work. HZ analyzed the sequencing data and wrote part of the manuscript. YQ and SJ revised the manuscript. WZ and JW collected the samples and clinical data. YK supervised the project and revised manuscript. All authors approved the final manuscript.

## Funding

This work was supported by the Natural Science Foundation of Shanghai (19ZR1476100), Science and Technology Commission of Shanghai Municipality (17JC1400804). Medical Engineering Cross Fund (YG2019GD02, YG2019QNB23, YG2019QNA49, and YG2019QNA52) and Laboratory Innovative Research Program of Shanghai Jiao Tong University (JCZXSJB2019002).

## Conflict of Interest

The authors declare that the research was conducted in the absence of any commercial or financial relationships that could be construed as a potential conflict of interest.
