# Supplementary material for: Post-Transcriptional Dysregulation of microRNA and Alternative Polyadenylation in Colorectal Cancer
Source: Front Genet. 2020 Feb 21;11:64. doi: 10.3389/fgene.2020.00064 (PMC7047281; doi:10.3389/fgene.2020.00064)
Supplement: Supplementary file 1 [file DataSheet_1.docx]

Supplementary Material

# Supplementary Figures and Tables

## Supplementary Figures


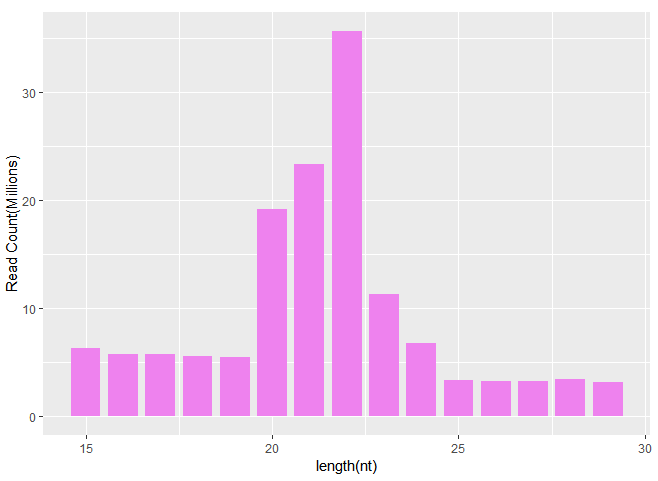


**Figure S1.** Length distribution of small RNA reads.


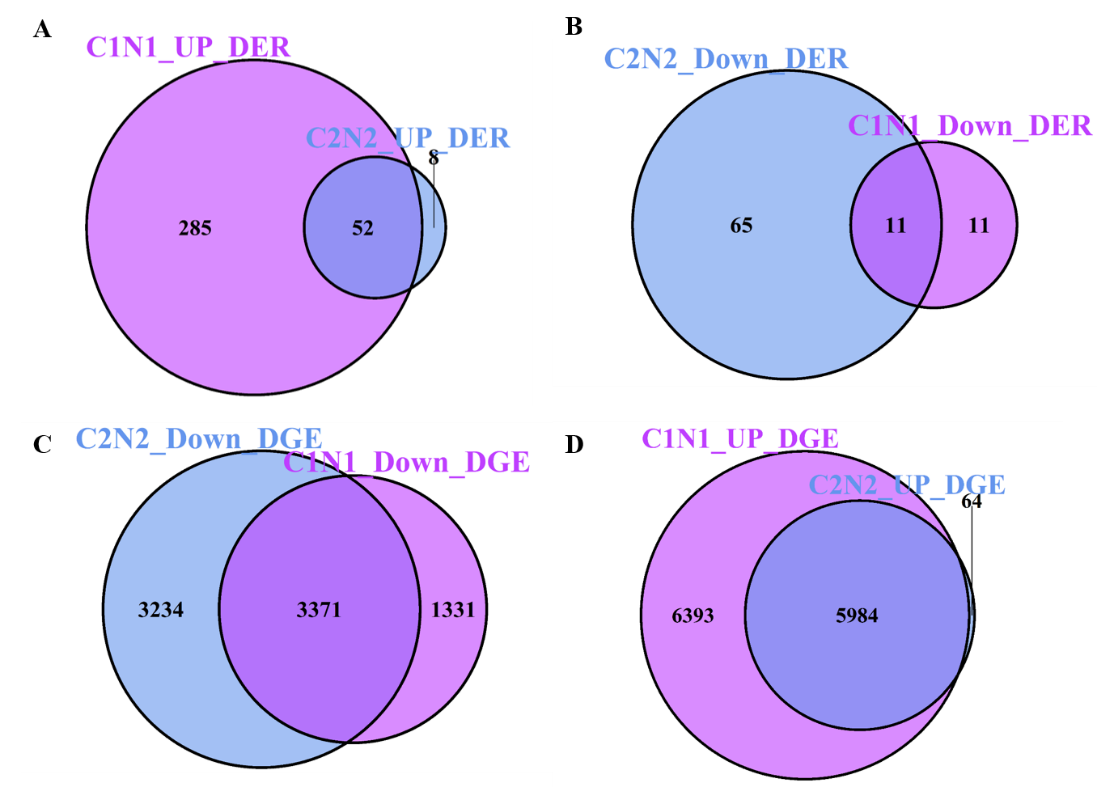


**Figure S2.** Significant differentially expressed miRNA and their targeted genes between CRC and paired normal tissues. (C: CRC tissue; N:paired normal tissue)

(A) Venn diagram of upregulated DERs between patients. (B) Venn diagram of Downregulated DERs between patients. (C) Venn diagram of predicted genes of downregulated DERs between patients. (D) Venn diagram of predicted genes of upregulated DERs between patients.


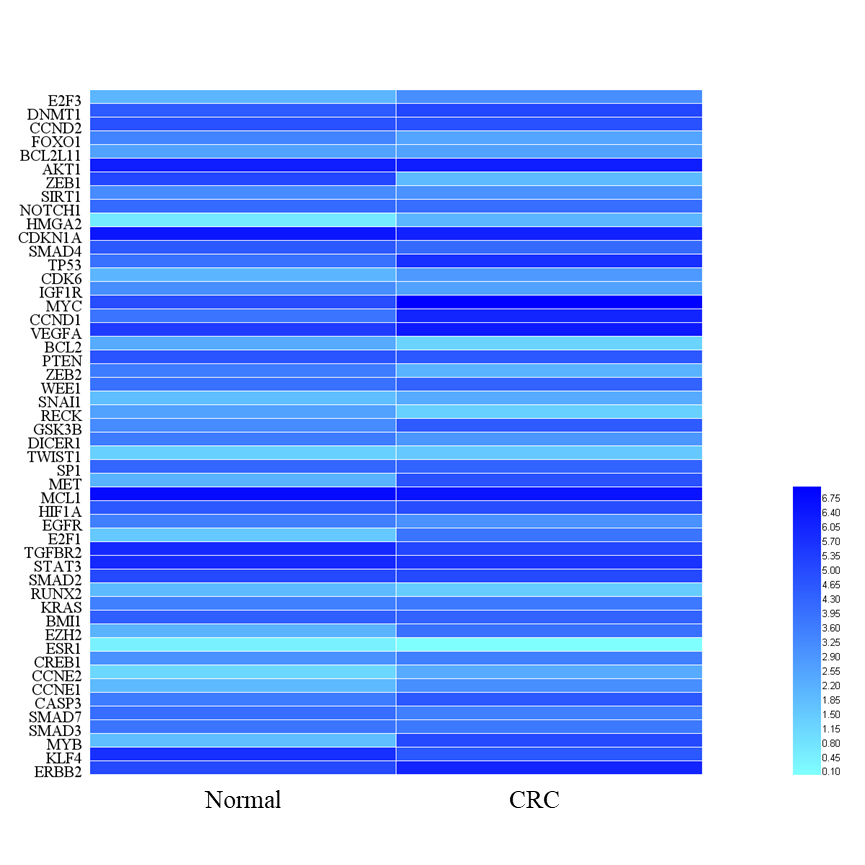


**Figure S3**. Heatmap of expression of Top 50 DERs target genes in CRC patients.


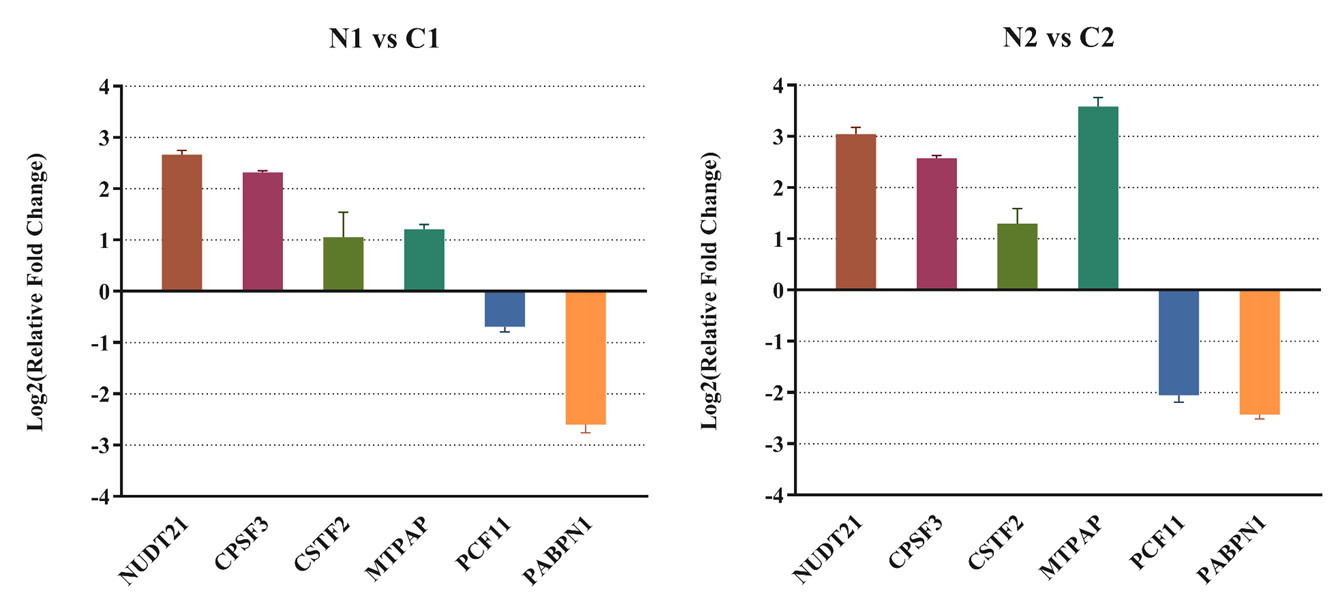


**Figure S4.** The qPCR results of NUDT21, CPSF3, CSTF2, MTPAP, PCF11 and PABPN1 expression in C1, N1, C2 and N2 samples

**
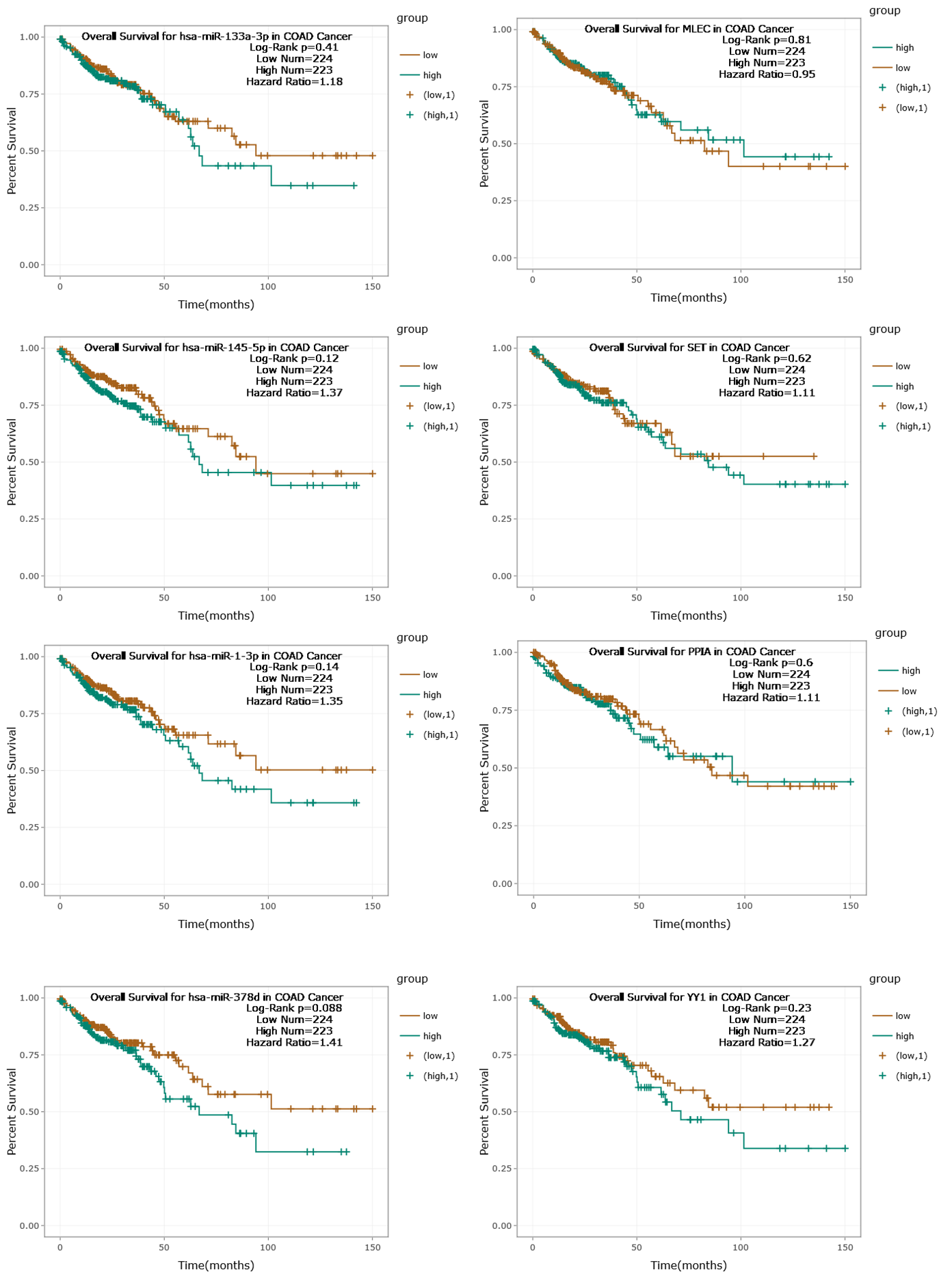
****Figure S5.** Survival Analysis for hsa-miR-133a-3p and *MLEC*, hsa-miR-145-5p and *SET*, hsa-miR-1-3p and *PPIA*, and hsa-miR-378d and *YY1*

## Supplementary Tables

**Table S1.Pathology information of clinical samples**

| Analysis Type | **Number** | **Gender** | **Age** | **Location** | | **Histopathological type** | **Size** | **Stage** | **Differentiation degree** |
| --- | --- | --- | --- | --- | --- | --- | --- | --- | --- |
| miRNA-seq | 1 | Male | 54 | Rectum | Adenocarcinoma | | 7.5×5.5×1.3 | T4N1M0 | Moderately to poorly differentiated |
|  | 2 | Female | 73 | Rectum | Adenocarcinoma | | 5×4.5×1.5 | T4N1M0 | Well differentiated |
| 3T-seq | 3 | Female | 82 | Right | Adenocarcinoma | | 6×5×1.2 | T4N2M0 | Poorly differentiated |
|  | 4 | Female | 56 | Rectum | Adenocarcinoma | | 5×4×4.5 | T4N2M0 | Moderately to poorly differentiated |
|  | 5 | Female | 73 | Rectum | Adenocarcinoma | | 5×4.5×1.5 | T4N1M0 | Well differentiated |

**Table S2. Detailed information on the sequencing miRNA library of CRC tissues (C1 and C2) and paired normal tissues (N1 and N2).**

| Sample | C1 | C2 | N1 | N2 |
| --- | --- | --- | --- | --- |
| Total Raw Reads | 26,847,093 | 78,973,651 | 37,988,667 | 105,352,122 |
| Total Raw Bases | 2,039,109,591 | 5,994,343,675 | 2,883,155,396 | 7,987,519,556 |
| Total Clean Reads | 26,045,542 | 70,296,157 | 33,831,747 | 85,486,392 |
| Total Clean Bases | 771,040,715 | 1,821,849,744 | 796,635,500 | 2,408,188,337 |
| Mapped Reads | 25,319,622 | 69,050,055 | 31,872,990 | 83,260,697 |
| Mapped Ratio | 97.21% | 98.23% | 94.21% | 97.40% |
| Unique Mapped Reads | 2,755,638 | 15,317,255 | 17,910,819 | 26,791,336 |
| Unique Mapped Ratio | 10.58% | 21.79% | 52.94% | 31.34% |

**Table S3. Identified known miRNA.**

**(Presented in the form of excel)**

**Table S4. Identified novel miRNA.**

**(Presented in the form of excel)**

**Table S5. CRC related DERs and DER-Target Genes.**

**(Presented in the form of excel)**

**Table S6. Function analysis.**

**(Presented in the form of excel)**

**Table S7.** **Detailed information on DER-targeted polyadenylation factors**

| **Type of APA factor** | **Gene Name** | **Official Full Name** | **Also known as** | **miRNA** | **miRNA Expression in CRC** |
| --- | --- | --- | --- | --- | --- |
| **cleavage and polyadenylation specific factor** | *CPSF1* | cleavage and polyadenylation specific factor 1 | CPSF160; P/cl.18; HSU37012 | hsa-miR-17-5p | Upregulated |
|  | *CPSF1* | cleavage and polyadenylation specific factor 1 | CPSF160; P/cl.18; HSU37012 | hsa-miR-1-3p | Downregulated |
|  | *CPSF3* | cleavage and polyadenylation specific factor 3 | CPSF73; CPSF-73 | hsa-miR-1-3p | Downregulated |
|  | *NUDT21* | nudix hydrolase 21 | CPSF5；CFIM25 | hsa-miR-1-3p | Downregulated |
|  | *CPSF3* | cleavage and polyadenylation specific factor 3 | CPSF73; CPSF-73 | hsa-miR-708-5p | Upregulated |
|  | *CPSF7* | cleavage and polyadenylation specific factor 7 | CFIm59 | hsa-miR-424-5p | Upregulated |
| **cleavage stimulation factor** | *CSTF2* | cleavage stimulation factor subunit 2 | CstF-64 | hsa-miR-224-5p | Upregulated |
|  | *CSTF3* | cleavage stimulation factor subunit 3 | CstF-77 | hsa-miR-1-3p | Downregulated |
| **cleavage and polyadenylation factor** | *PCF11* | PCF11 cleavage and polyadenylation factor subunit | — | hsa-miR-490-3p | Downregulated |
| **poly(A) binding protein** | *PABPN1* | poly(A) binding protein nuclear 1 | OPMD; PAB2; PABII; PABP2; PABP-2 | hsa-let-7i-3p | Upregulated |
| **symplekin protein** | *SYMPK* | symplekin | SYMPK | hsa-miR-1-3p | Downregulated |
| **poly(A) polymerase** | *TUT1* | terminal uridylyl transferase 1, U6 snRNA-specific | PAPD2;RBM21; URLC6;TUTase; STARPAP | hsa-miR-17-5p | Upregulated |
|  | *TUT1* | terminal uridylyl transferase 1, U6 snRNA-specific | PAPD2;RBM21; URLC6;TUTase; STARPAP | hsa-miR-183-5p | Upregulated |
|  | *TUT1* | terminal uridylyl transferase 1, U6 snRNA-specific | PAPD2;RBM21; URLC6;TUTase; STARPAP | hsa-miR-18a-5p | Upregulated |
|  | *TUT1* | terminal uridylyl transferase 1, U6 snRNA-specific | PAPD2; RBM21; URLC6; TUTase; STARPAP | hsa-miR-503-5p | Upregulated |
|  | *MTPAP* | mitochondrial poly(A) polymerase | PAPD1; SPAX4 | hsa-miR-17-5p | Upregulated |
|  | *MTPAP* | mitochondrial poly(A) polymerase | PAPD1; SPAX4 | hsa-miR-20a-5p | Upregulated |
|  | *MTPAP* | mitochondrial poly(A) polymerase | PAPD1; SPAX4 | hsa-miR-455-3p | Upregulated |
|  | *PAPD4* | poly(A) RNA polymerase D4, non-canonical | GLD2; TUT2 | hsa-miR-139-5p | Downregulated |
|  | *PAPD4* | poly(A) RNA polymerase D4, non-canonical | GLD2; TUT2 | hsa-miR-148b-3p | Upregulated |
|  | *PAPD4* | poly(A) RNA polymerase D4, non-canonical | GLD2; TUT2 | hsa-miR-152-3p | Upregulated |
|  | *PAPD4* | poly(A) RNA polymerase D4, non-canonical | GLD2; TUT2 | hsa-miR-301b-3p | Upregulated |

**Table S8. 3’UTR alteration and mRNA expression of DER-target genes.**

| miRNA | miRNA Expression change | Target Gene | Gene 3’UTR state | Gene Expression from TCGA database | *p-value* |
| --- | --- | --- | --- | --- | --- |
| hsa-miR-133a-3p | Down | *MLEC* | Shorten | Up | 0.0002 |
| hsa-miR-133a-3p | Down | *TMEM59* | Shorten | Down | <1E-12 |
| hsa-miR-135a-5p | Down | *MARCKS* | Shorten | Down | 0.0000 |
| hsa-miR-135a-5p | Down | *TXNIP* | Shorten | Down | <1E-12 |
| hsa-miR-139-5p | Down | *FOS* | Shorten | Down | 0.0625 |
| hsa-miR-139-5p | Down | *MLEC* | Shorten | Up | 0.0002 |
| hsa-miR-139-5p | Down | *TNPO1* | Shorten | Up | 0.0000 |
| hsa-miR-1-3p | Down | *ABI2* | Shorten | Up | 0.0000 |
| hsa-miR-1-3p | Down | *CALM1* | Shorten | Down | 0.0000 |
| hsa-miR-1-3p | Down | *CNN3* | Shorten | down | 0.1503 |
| hsa-miR-1-3p | Down | *DROSHA* | Lengthen | —— | —— |
| hsa-miR-1-3p | Down | *DSG2* | Lengthen | Down | 0.0003 |
| hsa-miR-1-3p | Down | *EML4* | Shorten | Up | 0.0000 |
| hsa-miR-1-3p | Down | *HSD17B11* | Shorten | Down | 0.0000 |
| hsa-miR-1-3p | Down | *IGFBP7* | Shorten | Up | 0.0000 |
| hsa-miR-1-3p | Down | *KIF5B* | Shorten | Up | 0.0000 |
| hsa-miR-1-3p | Down | *LONP2* | Shorten | Up | 0.0000 |
| hsa-miR-1-3p | Down | *MARCKS* | Shorten | Down | 0.0000 |
| hsa-miR-1-3p | Down | *NPTN* | Shorten | Down | 0.0000 |
| hsa-miR-1-3p | Down | *OXCT1* | Shorten | Down | 0.2160 |
| hsa-miR-1-3p | Down | *POLR2K* | Shorten | Up | 0.0000 |
| hsa-miR-1-3p | Down | *PPIA* | Shorten | Up | 0.0000 |
| hsa-miR-1-3p | Down | *RRBP1* | Shorten | Down | 0.0204 |
| hsa-miR-1-3p | Down | *SLC44A1* | Shorten | Down | 0.0000 |
| hsa-miR-1-3p | Down | *SRI* | Shorten | Down | 0.0000 |
| hsa-miR-1-3p | Down | *SRSF7* | Shorten | —— | —— |
| hsa-miR-1-3p | Down | *TIMP3* | Shorten | Up | 0.4244 |
| hsa-miR-145-5p | Down | *GOLM1* | Shorten | Down | <1E-12 |
| hsa-miR-145-5p | Down | *IVNS1ABP* | Shorten | Down | 0.0092 |
| hsa-miR-145-5p | Down | *KLF5* | Shorten | Down | 0.0443 |
| hsa-miR-145-5p | Down | *SET* | Shorten | Up | <1E-12 |
| hsa-miR-183-5p | UP | *CTDSPL* | Shorten | Down | 0.0000 |
| hsa-miR-183-5p | UP | *DGAT1* | Lengthen | Down | 0.1406 |
| hsa-miR-183-5p | UP | *GSPT1* | Shorten | Up | 0.0000 |
| hsa-miR-183-5p | UP | *RTN4* | Lengthen | Down | 0.0001 |
| hsa-miR-183-5p | UP | *TMED4* | Lengthen | Up | 0.0000 |
| hsa-miR-183-5p | UP | *TXNIP* | Shorten | Down | <1E-13 |
| hsa-miR-183-5p | UP | *USP22* | Shorten | Down | 0.0381 |
| hsa-miR-183-5p | UP | *YY1* | Shorten | Up | 0.0000 |
| hsa-miR-224-5p | UP | *NPTN* | Shorten | Down | 0.0000 |
| hsa-miR-224-5p | UP | *RBM3* | Lengthen | Up | 0.0000 |
| hsa-miR-224-5p | UP | *TXNIP* | Shorten | Down | <1E-14 |
| hsa-miR-335-3p | UP | *NT5DC1* | Shorten | Up | 0.0000 |
| hsa-miR-335-3p | UP | *SSR3* | Shorten | Up | 0.0000 |
| hsa-miR-378d | Down | *YY1* | Shorten | Up | 0.0000 |
| hsa-miR-424-3p | UP | *APP* | Shorten | Up | 0.0002 |
| hsa-miR-424-3p | UP | *BCLAF1* | Shorten | Up | 0.0000 |
| hsa-miR-424-5p | UP | *APP* | Shorten | Up | 0.0002 |
| hsa-miR-424-5p | UP | *CANX* | Shorten | Up | 0.0000 |
| hsa-miR-424-5p | UP | *CCND2* | Shorten | Up | 0.0000 |
| hsa-miR-424-5p | UP | *CTDSPL* | Shorten | Down | 0.0000 |
| hsa-miR-424-5p | UP | *HNRNPA2B1* | Lengthen | Up | <1E-12 |
| hsa-miR-424-5p | UP | *IVNS1ABP* | Shorten | Down | 0.0092 |
| hsa-miR-424-5p | UP | *KIF5B* | Shorten | Up | 0.0000 |
| hsa-miR-424-5p | UP | *NUCKS1* | Shorten | Up | 0.0000 |
| hsa-miR-424-5p | UP | *PDIA6* | Shorten | Up | 0.0000 |
| hsa-miR-424-5p | UP | *RTN4* | Lengthen | Down | 0.0001 |
| hsa-miR-424-5p | UP | *TBRG1* | Shorten | Up | 0.0130 |
| hsa-miR-424-5p | UP | *TXNIP* | Shorten | Down | <1E-15 |
| hsa-miR-431-5p | UP | *CANX* | Shorten | Up | 0.0000 |
| hsa-miR-431-5p | UP | *GFPT1* | Shorten | Down | 0.5801 |
| hsa-miR-431-5p | UP | *IRF2BP2* | Shorten | Up | 0.0000 |
| hsa-miR-431-5p | UP | *RBM3* | Lengthen | Up | 0.0000 |
| hsa-miR-431-5p | UP | *YY1* | Shorten | Up | 0.0000 |
| hsa-miR-450b-5p | UP | *CALM1* | Shorten | Down | 0.0000 |
| hsa-miR-450b-5p | UP | *IMPAD1* | Shorten | Up | 0.0000 |
| hsa-miR-450b-5p | UP | *MRPS10* | Shorten | Up | 0.0000 |
| hsa-miR-450b-5p | UP | *TMEM59* | Shorten | Down | <1E-13 |
| hsa-miR-490-3p | Down | *MRPS10* | Shorten | Up | 0.0000 |
| hsa-miR-490-3p | Down | *TMEM59* | Shorten | Down | <1E-14 |
| hsa-miR-490-5p | Down | *APP* | Shorten | Up | 0.0002 |
| hsa-miR-490-5p | Down | *FOS* | Shorten | Down | 0.0625 |
| hsa-miR-503-5p | UP | *CANX* | Shorten | Up | 0.0000 |
| hsa-miR-503-5p | UP | *CCND2* | Shorten | Up | 0.0000 |
| hsa-miR-503-5p | UP | *CTDSPL* | Shorten | Down | 0.0000 |
| hsa-miR-503-5p | UP | *KIF5B* | Shorten | Up | 0.0000 |
| hsa-miR-503-5p | UP | *NUCKS1* | Shorten | Up | 0.0000 |
| hsa-miR-503-5p | UP | *RTN4* | Lengthen | Down | 0.0001 |
| hsa-miR-542-5p | UP | *PPP1CB* | Shorten | Down | <1E-12 |
| hsa-miR-655-3p | UP | *CANX* | Shorten | Up | 0.0000 |
| hsa-miR-655-3p | UP | *KIF5B* | Shorten | Up | 0.0000 |
| hsa-miR-655-3p | UP | *PTP4A1* | Shorten | Down | 0.0000 |
| hsa-miR-708-5p | UP | *TMED4* | Lengthen | Up | 0.0000 |
| hsa-miR-708-5p | UP | *USP9X* | Shorten | Up | 0.0011 |
| hsa-miR-96-5p | UP | *CCND2* | Shorten | Up | 0.0000 |

**Table S9. Detailed information on DERs and DER-target genes related to APA-mediated 3’UTR alternation in CRC.**

| miRNA | miRNA expression in CRC | Mature sequence | Target gene | Full names | 3’ UTR state | miRNA binding site loss | Gene expression in CRC |
| --- | --- | --- | --- | --- | --- | --- | --- |
| hsa-miR-133a-3p | Downregulation | UUUGGUCCCCUUCAACCAGCUG | *MLEC* | malectin | Shorten | 1/1 | Upregulation |
| hsa-miR-145-5p | Downregulation | GUCCAGUUUUCCCAGGAAUCCCU | *SET* | SET nuclear proto-oncogene | Shorten | 1/1 | Upregulation |
| hsa-miR-1-3p | Downregulation | UGGAAUGUAAAGAAGUAUGUAU | *PPIA* | peptidylprolyl isomerase A | Shorten | 2/3 | Upregulation |
| hsa-miR-378d | Downregulation | ACUGGACUUGGAGUCAGAAA | *YY1* | YY1 transcription factor | Shorten | 3/3 | Upregulation |
